# Reducing the Inappropriate Use of Proton Pump Inhibitors in an Internal Medicine Residency Clinic

**DOI:** 10.7759/cureus.6609

**Published:** 2020-01-09

**Authors:** Joshua Boster, Lacy E Lowry, Matthew L Bezzant, Brandon Kuiper, Luke Surry

**Affiliations:** 1 Internal Medicine, Brooke Army Medical Center, Fort Sam Houston, USA; 2 Internal Medicine, San Antonio Uniformed Services Health Education Consortium/Brooke Army Medical Center, San Antonio, USA; 3 Internal Medicine, Brooke Army Medical Center, San Antonio, USA; 4 Internal Medicine, San Antonio Uniformed Services Health Education Consortium, San Antonio, USA

**Keywords:** ppi

## Abstract

Introduction

Proton pump inhibitors (PPI) are commonly prescribed in the primary care setting. While generally considered to be safe, there is growing evidence suggesting that PPI misuse is associated with a variety of significant adverse outcomes and unnecessary cost. The goal of this quality improvement project was to identify patients with non-guideline recommended PPI prescriptions in our internal medicine residency clinics and implement a process to de-prescribe or reduce the dose of PPIs across this patient population.

Methods

PPI prescription rates, dosage, and indication were extracted from the medical records of all 854 patients empaneled to the internal medicine residency clinics at a multicenter closed referral military hospital system. Appropriate PPI indication was consensus based upon published guidelines, and patients without an appropriate indication were targeted for intervention. These patients were directly contacted by their primary care physicians, via phone or during a clinic visit, to discuss the risks and benefits of ongoing PPI use as well as alternative therapies or tapering regimens at the physician’s discretion. For moderate to high dose PPI, the dose was decreased by 50% every week until the lowest tolerated dose was achieved or until discontinuation. For low dose PPI, discontinuation was recommended as the initial intervention. Six months following the intervention, the empanelment was reevaluated for ongoing PPI usage, tapered dosage, or discontinuation.

Results

Of a total of 854 patient records reviewed at the initiation of the project, 322 patients were noted to be prescribed PPIs. Of this subset, 66% (217/322) did not meet a guideline recommended indication for their use. At the completion of the six-month intervention period, 44% (96/217) of patients were successfully weaned to a reduced dose or were no longer using a PPI.

Conclusions

PPIs are widely used and generally considered to be a well-tolerated therapy for acid-secretion disorders. PPI overprescription and the associated adverse effects and economic burden are increasingly recognized. We show that a simple, focused, resident-driven quality improvement intervention can be effective in de-prescribing efforts to reduce inappropriate PPI use in the outpatient primary care setting.

## Introduction

Proton pump inhibitors (PPIs) are the most widely prescribed classes of medications in the United States [1]. PPIs remain the mainstay of treatment for a variety of gastrointestinal diseases such as gastroesophageal reflux disease (GERD), dyspepsia, and peptic ulcer disease. However, there is increasing concern that PPIs are being overutilized in multiple treatment areas including the ambulatory care setting [2]. This overutilization has significant economic implications in the United States accounting for more than $10 billion in estimated annual healthcare costs [3]. Additionally, there is a growing body of evidence suggesting that overutilization and long-term inappropriate use of PPIs are associated with adverse outcomes including pneumonia, *Clostridioides difficile* infection, increased risk of bone fractures, and drug interactions [4-6]. In order to prevent the adverse outcomes associated with overuse or misuse of PPIs and to avoid medical waste, it is essential to implement interventions to improve guideline-directed use of PPIs.

The significant prevalence of non-evidence based PPI prescriptions has been attributed to the high incidence of acid-related disease, high efficacy, and relatively low toxicity profile of PPIs, along with widespread over-the-counter availability and low cost [7]. For certain conditions such as Barrett’s esophagus and severe esophagitis, indefinite PPI therapy may be indicated; however, for indications such as GERD, *Helicobacter pylori* infection, and peptic ulcer disease, the recommended duration of PPI therapy is limited to 2-12 weeks [8]. Despite a lack of indication for indefinite PPI therapy, patients are often continued on PPI therapy indefinitely without reevaluation for symptom improvement or resolution in the ambulatory setting. This contributes to polypharmacy with the associated risk of adverse reactions and medication errors. Although PPI misuse in the ambulatory setting is common, with a prior report of inappropriate PPI prescription rates of 36.1%, highly variable success rates (14%-64%) for discontinuation have been published, suggesting difficulty in discontinuing these medications following initiation [9-14].

Our resident-led quality improvement initiative had two aims: (1) to assess how common PPIs are prescribed without clear indication in our internal medicine clinics, and (2) to effectively discontinue or taper inappropriate PPI prescriptions. 

## Materials and methods

PPI prescription rates, dosage, and indication were abstracted from all 854 patients empaneled to the internal medicine residency clinics at a multi-center closed referral hospital system. Appropriate PPI indication was based upon consensus-driven indications (Table 1), and patients without an appropriate indication were targeted for intervention [15].

**Table 1:** Consensus-based appropriate indications for prolonged PPI use PPI, proton pump inhibitor; GI, gastrointestinal; NSAID, non-steroidal anti-inflammatory drug; UGIB, upper gastrointestinal bleed

Appropriate indications for PPI use > 8 weeks duration
Prior GI bleed
Barrett’s esophagus
Los Angeles grade D esophagitis
Ongoing NSAID use
Dual-antiplatelet therapy (with prior UGIB, or other risk factor)
Active hypersecretory condition

**Figure 1:**
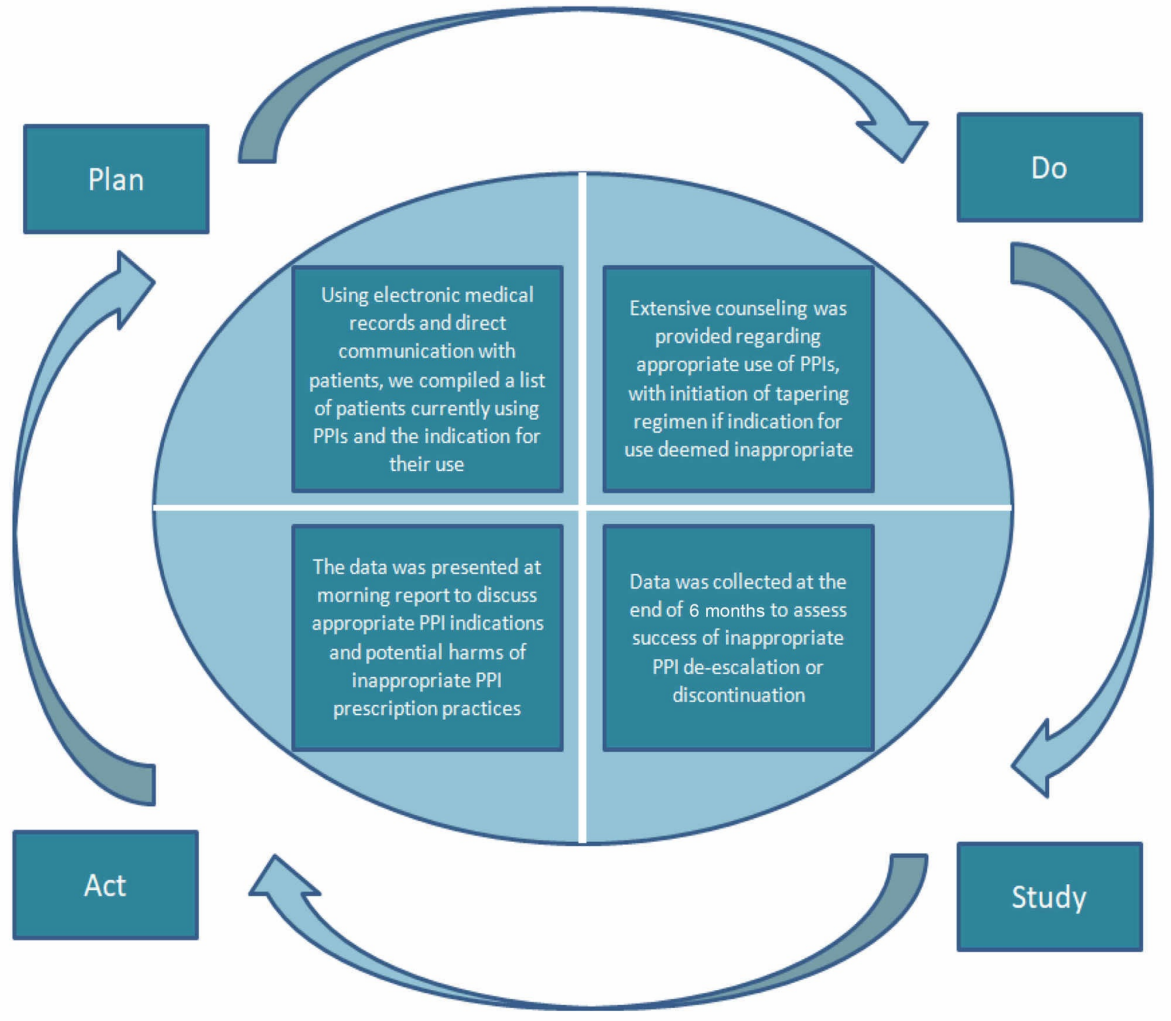
PDSA cycle for discontinuing or tapering inappropriate PPI prescriptions PDSA, Plan-Do-Study-Act; PPI, proton pump inhibitor

A Plan-Do-Study-Act (PDSA) cycle was designed to implement and evaluate the planned intervention, with the goal of discontinuing or tapering inappropriate PPI prescriptions (Figure 1).

These patients were directly contacted by their resident physicians, via phone or during a clinic visit, to discuss the risks and benefits of ongoing PPI use as well as alternative therapies or tapering regimens at the physician’s discretion. For moderate to high dose PPI, the dose was decreased by 50% every week until the lowest tolerated dose was achieved or until discontinuation. For low dose PPI, discontinuation was recommended as the initial intervention. Six months following the intervention, the empanelment was reevaluated for ongoing PPI usage, tapered dosage, and discontinuation.

## Results

**Figure 2:**
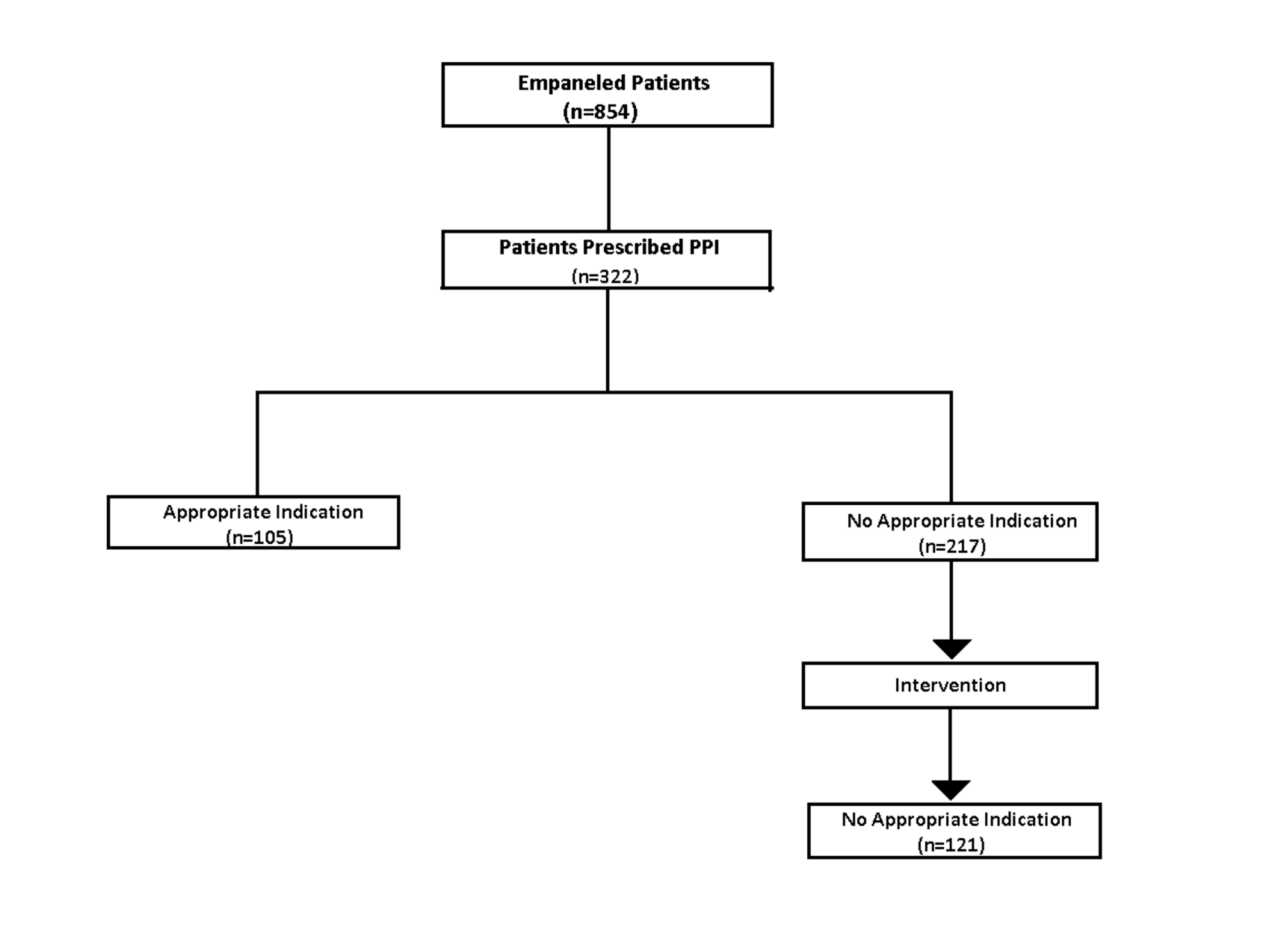
Summary of study process and outcome n, number; PPI, proton pump inhibitor

Of a total of 854 patient records reviewed at the initiation of the project, 322 patients were noted to be using PPIs. Of this subset, 217 (67%) did not meet a guideline recommended indication for their use. At the completion of the six-month intervention period, 44% (96/217) of patients were successfully weaned to a reduced dose or no longer using a PPI (Figure 2).

## Discussion

When properly utilized PPIs have provided immense benefit to patients since their release in the 1990s, but the degree of PPI misuse and its potential consequences have only recently emerged. Our project revealed the prevalence of inappropriate PPI prescriptions in our patient cohort to be nearly twice that (67% vs. 36%) cited in previous general population studies. Reasons for this are unclear, but may be attributed to increased access to healthcare in our population as well as negligible or no co-pays for prescription and non-prescription medications and automaticity of prolonged prescription courses. The percentage of patients who either decreased their PPI dosage or discontinued PPI use in our study was within the mid-to-upper limit of the previously cited range (44% vs. 14%-64%). However, it remains unclear the number of patients who required uptitration of PPIs following completion of the intervention. Although our results suggest a moderately successful intervention, several significant limitations in the project must be acknowledged. First, this intervention involved a small sample of military-affiliated patients with ready access to healthcare, potentially limiting our ability to extrapolate these results to the general population. Second, we did not stratify patients by starting dose, nor did we delineate the number of patients who completely stopped taking PPIs vs. those who were tapered. Finally, and arguably most significantly, we did not incorporate a systematic change that would allow sustainability of our intervention over time, instead focusing on provider/patient education during this particular period alone.

In spite of these limitations, the promising nature of our results indicates utility in re-doing the project with a more systematic approach in mind. This would require a multidisciplinary team of clinicians, pharmacists, and ancillary staff to ensure that all stakeholders are engaged. Aside from provider and patient education, potential system-based interventions include placing a lock on first time PPI dosing to allow for only a total of eight weeks of therapy, or setting a reminder which displays established indications for long-term PPI use with each prescription renewal.

While the small sample size and aforementioned limitations of our project restrict conclusions regarding morbidity and mortality benefit, the cost-benefit to our system is undeniable. Omeprazole and pantoprazole are the two most commonly prescribed PPIs, with a cost of $0.054/20 or $0.099/40 mg tablet and $0.1259/20 or 40 mg tablet, respectively. Using pantoprazole as an example, one tablet of either dosage for one patient per year is approximately $46 ($0.1259 x 365 days). Thus, 100 patients unnecessarily receiving pantoprazole is approximately $4600 per year. If 67% of our patient population is incorrectly prescribed PPIs, this sum is significant. Additionally, this does not account for management of potential downstream consequences of PPI use, such as pneumonia, *Clostridioides difficile* infections, or osteoporosis. 

## Conclusions

PPI overutilization is a common problem with potential for serious associated adverse patient outcomes and significant cost. Although discontinuation of PPIs in the ambulatory setting is difficult, it is feasible, and resident-led quality improvement initiatives are an effective strategy for reducing inappropriate PPI prescriptions in this setting.
